# Analysis of metabolic differences in maize in different growth stages under nitrogen stress based on UPLC-QTOF-MS

**DOI:** 10.3389/fpls.2023.1141232

**Published:** 2023-04-03

**Authors:** Guipei Song, Yanli Lu, Yuhong Wang, Caie Nie, Mengze Xu, Lei Wang, Youlu Bai

**Affiliations:** State Key Laboratory of Efficient Utilization of Arid and Semi-arid Arable Land in Northern China/Key Laboratory of Plant Nutrition and Fertilizer, Ministry of Agriculture and Rural Affairs/Institute of Agricultural Resources and Regional Planning, Chinese Academy of Agricultural Sciences, Beijing, China

**Keywords:** maize, nitrogen, UPLC-QTOF-MS, metabolomics, secondary metabolism

## Abstract

**Introduction:**

Maize has a high demand for nitrogen during the growth period. The study of metabolic changes in maize can provide a theoretical basis for rational nitrogen nutrition regulation.

**Methods:**

In order to investigate the changes of different metabolites and their metabolic pathways in maize leaves under nitrogen stress, we used ultra-performance liquid chromatography coupled with quadrupole time-of-flight mass spectrometry (UPLC-QTOF-MS) for metabolomic analysis of maize leaves under different nitrogen treatments at three critical growth stages (V4, V12 and R1) in a pot experiment under natural conditions.

**Results and discussion:**

The results showed that nitrogen stress significantly affected sugar metabolism and nitrogen metabolism, and affected carbon and nitrogen balance, and the effects of stress on maize leaves metabolism increased with the growth process. Metabolic pathways such as the TCA cycle and starch and sucrose metabolism were mainly affected at the seeding stage (V4). The stress response to nitrogen deficiency also showed significant upregulation of flavonoids such as luteolin and astragalin during the booting stage (V12) and anthesis-silking stage (R1). During R1 stage, the synthesis of tryptophan and phenylalanine and the degradation of lysine were significantly affected. Compared with nitrogen stress, the metabolic synthesis of key amino acids and jasmonic acid were intensified and the TCA cycle was promoted under nitrogen sufficiency conditions. This study initially revealed that the response mechanism of maize to nitrogen stress at the metabolic level.

## Introduction

Metabolomics is a systems biology discipline that was developed after genomics and proteomics (Hall et al., 2002; Nicholson et al., 2002; Kusano et al., 2011; Turner et al., 2016). There are more than 200,000 metabolites in plants, including primary metabolites that are essential for maintaining plant life and growth and development, and secondary metabolites that are closely related to plant disease and stress resistance, which are generated using primary metabolites (Pichersky and Gang, 2000). Although these metabolites are diverse, they are all interrelated. Moreover, as the terminal of the material basis of biological events, the changes in plant metabolites relatively closely representative of the biological phenotype and directly and accurately reflect the physiological state of plants (Fiehn, 2002; Schauer and Fernie, 2006; Shulaev et al., 2008).

As an important food crop, a major fodder crop and an efficient cash crop, maize plays a very important role in agricultural development and food security (Mi et al., 2010; Zhai et al., 2022). Maize has a high demand for nitrogen during the growing period, and nitrogen, as a basic element for normal plant growth and development, is a component of amino acids, proteins, chlorophyll, nucleic acids, and some coenzyme factors in plants, and plays an important role in plant metabolism. Reasonable regulation of nitrogen fertilization is not only beneficial to food security but also an important guarantee of ecological and environmental security (Hirel et al., 2001; Liu et al., 2018). Studies have shown that the amino acid content in the leaves of crops such as tobacco (Fritz et al., 2006), tomato (Urbanczyk-Wochniak and Fernie, 2005) and *Arabidopsis* (Tschoep et al., 2009; Krapp et al., 2011) tends to decrease under nitrogen deficiency, and sugars and secondary metabolites tend to increase. Schlüter et al. (2012) found a small accumulation of starch and a corresponding increase in carbohydrate content in maize leaves after low nitrogen stress treatment. The response of plants to stress is a complex life process. A large number of metabolic intermediates and end products are produced in the whole regulatory network. Metabolomics provides us with technical and analytical means to understand the differences in crop metabolites under abiotic stress and the key metabolic pathways that are regulated (Shulaev et al., 2008; Xin et al., 2019). This provides a new method to understand the mechanisms underlying the nitrogen utilization response in maize.

Changes in metabolites (groups) under nitrogen stress are influenced by the cultivation environment (Krapp et al., 2011; Schlüter et al., 2012) and duration of stress (Amiour et al., 2012). Previous studies on abiotic stress metabolomics have mostly focused on materials (such as seedlings) cultured under controlled conditions (Yang et al., 2017; Cao et al., 2020). In this study, we used ultra-performance liquid chromatography coupled with quadrupole time-of-flight mass spectrometry (UPLC-QTOF-MS) for metabolomic analysis of maize leaves under different nitrogen treatments at three critical growth stages (V4, V12 and R1) in a pot experiment under natural conditions to identify and screen out differential metabolites with distinctive characteristics, with the goal of revealing the response mechanism of maize to nitrogen stress through the differential metabolites and their metabolic pathways in different fertility periods under natural conditions and to provide a scientific basis for the diagnosis and regulation of nitrogen stress during the maize growing period.

## Materials and methods

### Experimental design

The pot experiment was conducted in the international agricultural high-tech industrial park of the Chinese Academy of Agricultural Sciences (Langfang City, Hebei Province, N39°35′47.03″, E116°35′16.24″) from June to October 2021. The test soil was taken from the deep soil in the park, and the soil type was tidal soil with sandy texture. The chemical properties of the test soil were determined by the ASI method by the National Center for Soil Testing and Fertilization, and the basic physicochemical properties are shown in **Table 1**. The test crop was summer maize ‘Zhengdan 958’, the test nitrogen fertilizer was urea (N = 46%), the phosphorus fertilizer was diammonium phosphate (N = 18%, P_2_O_5_ = 46%) and potassium dihydrogen phosphate (P_2_O_5_ = 51.5%, K_2_O = 33.5%), and the potassium fertilizer was potassium sulfate (K_2_O = 52%).

**Table 1:** The chemical properties of the experimental soil.

pH	Organic matter(%)	$NO_{3}^{-}$ –N(mg/kg)	$NH_{4}^{+}$ -N(mg/kg)	Olsen-P(mg/kg)	Available K(mg/kg)
8.30	0.32	3.11	1.27	4.37	43.30

Two levels of nitrogen application were set up, deficient nitrogen (DN) and sufficient nitrogen (SN), with nitrogen application rates of 0 and 540 kg/hm^2^, respectively; Phosphorus and potassium are supplied in sufficient amount with a phosphorus application (P_2_O_5_) rate of 360 kg/hm^2^, and a potassium (K_2_O) application rate of 300 kg/hm^2^. They were converted into 7.885 g/pot nitrogen, 5.257 g/pot phosphorus, and 4.381 g/pot potassium by the soil capacity of 1.13 g/cm^3^ on an air-dried weight basis. The test pots were 36 cm in diameter and 40 cm in height, filled with 33 kg of air-dried soil (passed through a 2 mm sieve), 40% of the nitrogen fertilizer and all of the phosphorus and potassium fertilizers were mixed with soil and potted, and the remaining 60% of the nitrogen fertilizer was applied with water after sampling at V12 stage.

### Plant materials and sample preparation

#### Sample collection and phenotypic index determination

With the growth process, the time of maize under nitrogen stress was increasing. Three key growth stages including V4, V12 and R1 were selected for sample collection. Plant height was measured using a tape measure. Plant height before the development of male ears was measured from the base of the stalk to the tip of the leaf after the corn leaves had been straightened; plant height following the development of male ears was measured from the base of the stalk to the tip of the male ears. Leaf SPAD values were obtained using a hand-held SPAD meter (SPAD-502, Minolta, Japan), and the SPAD value of the leaf was determined by selecting the upper, middle, and lower points of the latest fully expanded leaf/ear leaf and averaging them. The dry weight of the leaves was determined by the drying method. The whole fresh leaf samples were oven-dried for 30 minutes at 105°C and then heated at 70°C to a constant weight to determine the dry matter. The uppermost fully expanded leaves were collected at V4 and V12 stages, and the ear leaves were collected at R1 stage. Three replicates of each treatment were performed and the nitrate reductase (NR) and glutamine synthetase (GS) activities were measured by double antibody sandwich assay. Six replicates of each treatment were performed for metabolomics data determination, and the leaves were stored in liquid nitrogen immediately after being cutoff and brought to the laboratory for measurement. All samples from different treatments were mixed in equal amounts to prepare a sample for quality control.

#### Sample extraction

The plant tissues (80 mg leaves) were quickly frozen in liquid nitrogen immediately after collection and ground to a fine powder with a mortar and pestle. Then, the sample was added to a precooled methanol/acetonitrile/water solution (2:1:1, v/v), vortexed, sonicated at low-temperature (4°C) for 30 min, and left to stand at -20°C for 10 min. The sample was then centrifuged at 14000 rcf for 20 min at 4°C, the supernatant was vacuum dried, 100 µL of acetonitrile aqueous solution (acetonitrile: water = 1:1, v/v) was added for mass spectrometry analysis, the sample was vortexed and then centrifuged at 14000 rcf for 15 min at 4°C, and the supernatant was taken for analysis.

#### Chromatographic conditions

The samples were separated on an Agilent 1290 Infinity LC HILIC column, the column temperature was 25°C, the flow rate was 0.5 mL/min, the injection volume was 2 µL, mobile phase A was composed of water + 25 mM ammonium acetate + 25 mM ammonia, and mobile phase B was acetonitrile. The samples were placed in a 4°C autosampler throughout the analysis. To avoid the effects caused by the fluctuation of the instrument detection signal, a random order was used for the continuous analysis of the samples. QC samples were inserted in the sample queue for monitoring and evaluating the stability of the system and the reliability of the experimental data.

#### Mass spectrometry conditions

Mass spectrometry was performed with a Triple TOF 6600 mass spectrometer (AB SCIEX) using electrospray ionization (ESI) in positive and negative ionization modes. The ESI source setup parameters were as follows: nebulizing gas auxiliary heating gas 1 (Gasl), 60; auxiliary heating gas 2 (Gas2), 60; curtain gas (CUR), 30 psi; ion source temperature, 600°C; spray voltage (ISVF), ± 5500V (positive and negative modes); precursor mass-to-charge ratio detection range, 60-1000; product ion mass-to-charge ratio detection range, 25-1000; primary mass spectrometry scan accumulation time, 0.20 s/spectra; and secondary mass spectrometry scan accumulation time, 0.05 s/spectra. Secondary mass spectra were acquired in data-dependent acquisition (DDA) mode and in peak intensity value screening mode, with a declustering potential (DP) of ±60 V (positive and negative modes), a collision energy of 35 ± 15 eV, and DDA settings as follows: dynamic exclusion of isotopic ions range, 4 Da, and 10 acquisitions per scan.

### Data processing and multivariate analysis

The raw data in Wiff format was converted into “. mzXML” format by ProteoWizard, and then the peak alignment, retention time correction and peak area extraction were performed by MSDAIL software. The data extracted from MSDAIL were first identified by metabolite structure and data preprocessing, then the quality of experimental data was evaluated, and finally the data was analyzed. The quality of the model was tested with 7-fold cross validation, then the validity of the model was verified by cross-validation of R^2^Y (the interpretability of the model for categorical variable Y) and Q^2^ (predictability of the model). Finally, through the permutation test (randomly change the order of the categorical variables Y to obtain different random Q^2^ values) to further test the validity of the model. By OPLS-DA analysis and student’s t-test, we obtained two vital parameters, VIP (variable importance in projection) and *p* value. The value of VIP > 1 meanwhile *p* value < 0.05 were the standard for us to select the differential metabolites between every two groups. The metabolites were blasted against the online Kyoto Encyclopedia of Genes and Genomes (KEGG) database (http://geneontology.org/) and were subsequently mapped to pathways in KEGG. The differential expression fold change (FC) to indicate the up- and downregulation of substances, with FC > 1 indicating upregulation and FC < 1 indicating downregulation. KEGG pathway enrichment analysis takes the KEGG pathway as a unit, and analyzes and calculates the significance level of metabolic enrichment in each pathway through Fisher’s exact test to determine the metabolism and signal transduction pathways that are significantly affected. Excel 2018 and Adobe Illustrator 2021 were used to generate graphs.

## Results

### Effects of different nitrogen treatments on maize growth and development

**Figure 1:**
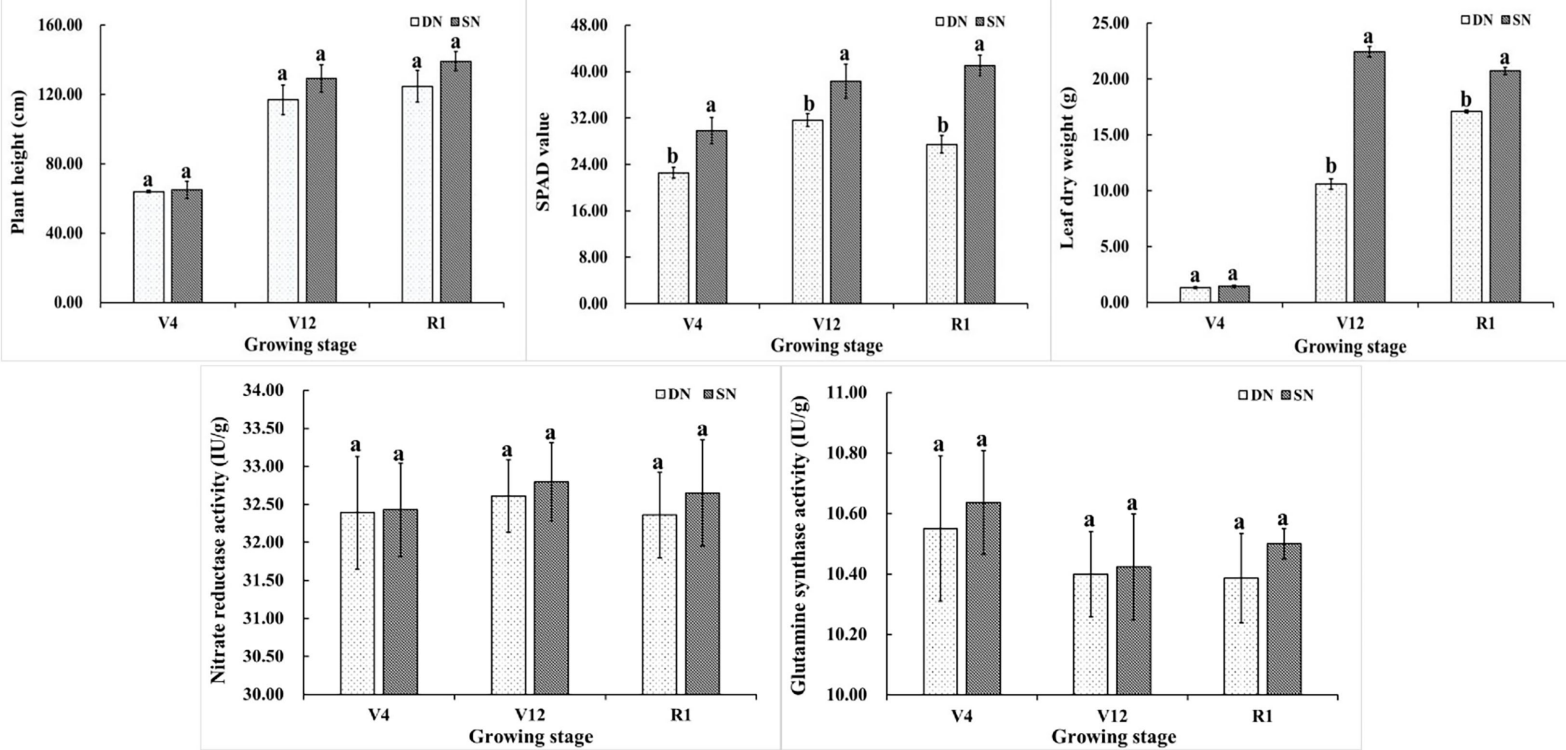
Plant height, SPAD value, leaf dry weight, NR and GS enzyme activities of maize under different treatments. (Note: Different letters above the bars indicate significant difference among treatments under the same nitrogen condition (P<0.05)).

**Figure 1** depicts the variations in plant height, SPAD, leaf dry weight, and the activity of two important enzymes involved in nitrogen metabolism (NR and GS) in maize grown under various nitrogen treatments. It can be seen from the figure that the plant height of maize shows an increasing trend during the growth period. The plant height increased rapidly from V4 to V12 and tended to be stable from V12 to R1. The plant height of maize under SN treatment was higher than that under DN treatment in different growth stages, and there was no significant difference between the two treatments. Nitrogen is the main component of chlorophyll, and the SPAD value represents the relative content of chlorophyll. The SPAD values under SN treatment were significantly higher than those under DN treatment. The SPAD values under DN treatment initially increased and then decreased as the maize growth process progressed. The SPAD values of leaves under SN treatment showed an increasing trend. Different nitrogen treatments at various growth stages resulted in distinct dry matter accumulations in leaves. At the V4 stage, there was no significant difference in leaf dry weight between the two nitrogen treatments. At V12 and R1 stages, the dry weight of leaves under SN treatment was significantly higher than that under DN treatment. The dry weight of leaves under DN treatment reaches its maximum value at the R1 stage. The dry weight of leaves under SN treatment reached its maximum value at V12 stage, but there was a small difference in the dry weight of leaves at R1 stage. As shown in **Figure 1**, the overall trend for NR enzyme activity was one of growing before declining, whereas the overall trend for GS enzyme activity was one of falling before rising. Between SN treatment and DN treatment, there was no significant difference in the two enzymes’ activities.

### Multivariate statistical analysis of metabolites under different nitrogen treatments

**Figure 2:**
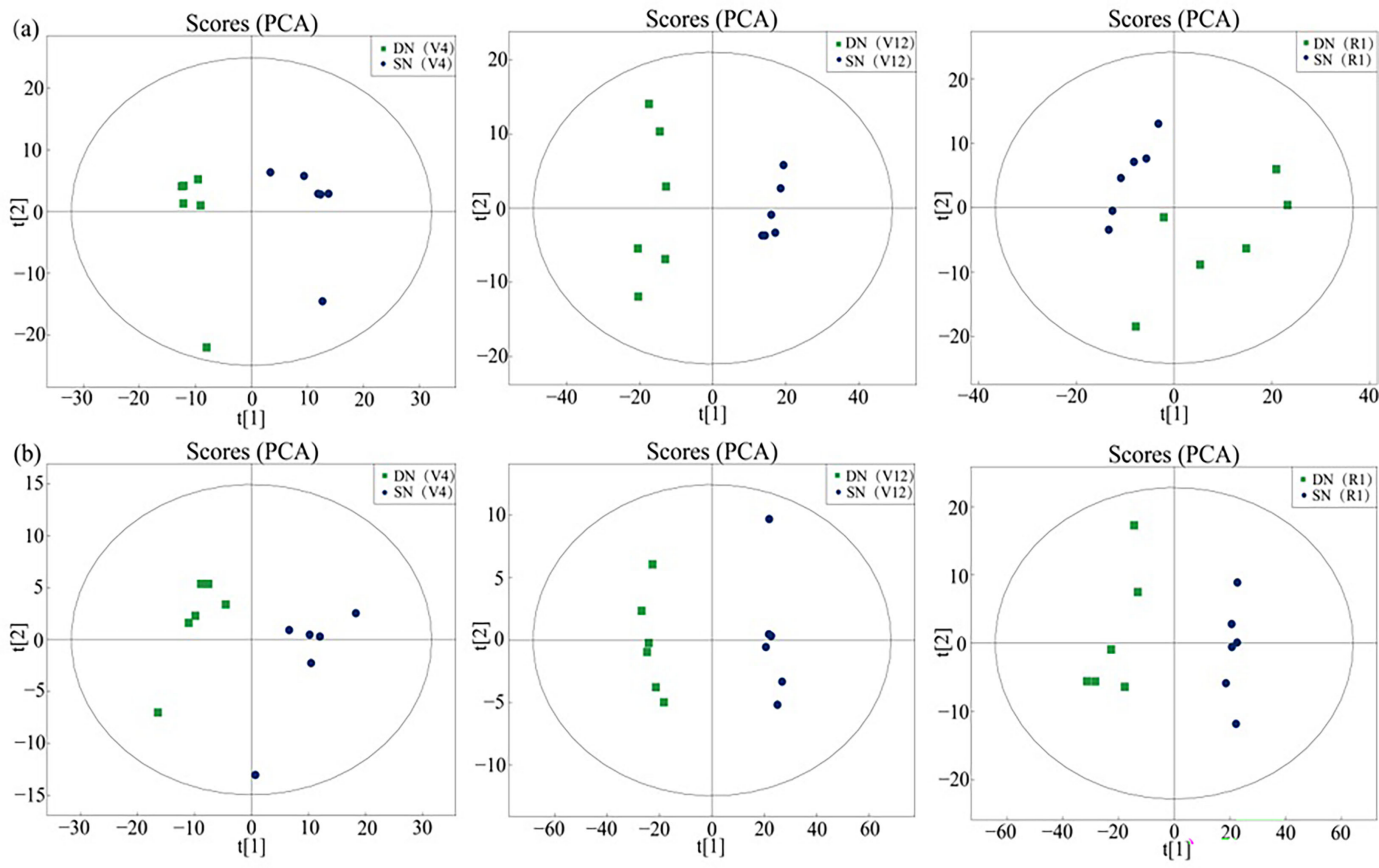
Sample score plot for the first (t [1]) and second (t [2]) principal components provided by PCA for the identified metabolites in *maize* leaf samples (DN, SN) in ESI^+^ mode **(A)** and ESI^−^ mode **(B)**. Each group is represented by six samples.

To reveal the response of maize leaves to different nitrogen application treatments, metabolites in the samples were detected by ultra-high-performance liquid chromatography-tandem time of flight mass spectrometry (UHPLC-Q-TOF MS), and metabolite profiles in ESI^+^ and ESI^-^ mode were analyzed by LC−MS/MS. By comparing DN and SN treatments, a total of 101 and 37 differential metabolites were detected in ESI^+^ and ESI^-^ modes at V4 stage, 128 and 57 differential metabolites were detected in ESI^+^ and ESI^-^ modes at V12 stage, and 120 and 38 differential metabolites were detected in ESI^+^ and ESI^-^ modes at R1 stage, respectively. The supplementary data sets contained all the metabolites detected in the two ion modes in this experiment. Detailed information about these metabolites was included. PCA revealed the following differences in metabolite profiles: different clusters were formed under different nitrogen application treatments (**Figure 2**). A total of 32.27%, 56.22% and 41.45% of the differences were explained by the first principal component in ESI^+^ mode (a in **Figure 2**), and 32.99%, 54.86% and 42.30% of the differences were explained by the first principal component in ESI^-^ mode (b in **Figure 2**), indicating a strong differential response of the nitrogen treatment at the metabolic level.

**Figure 3:**
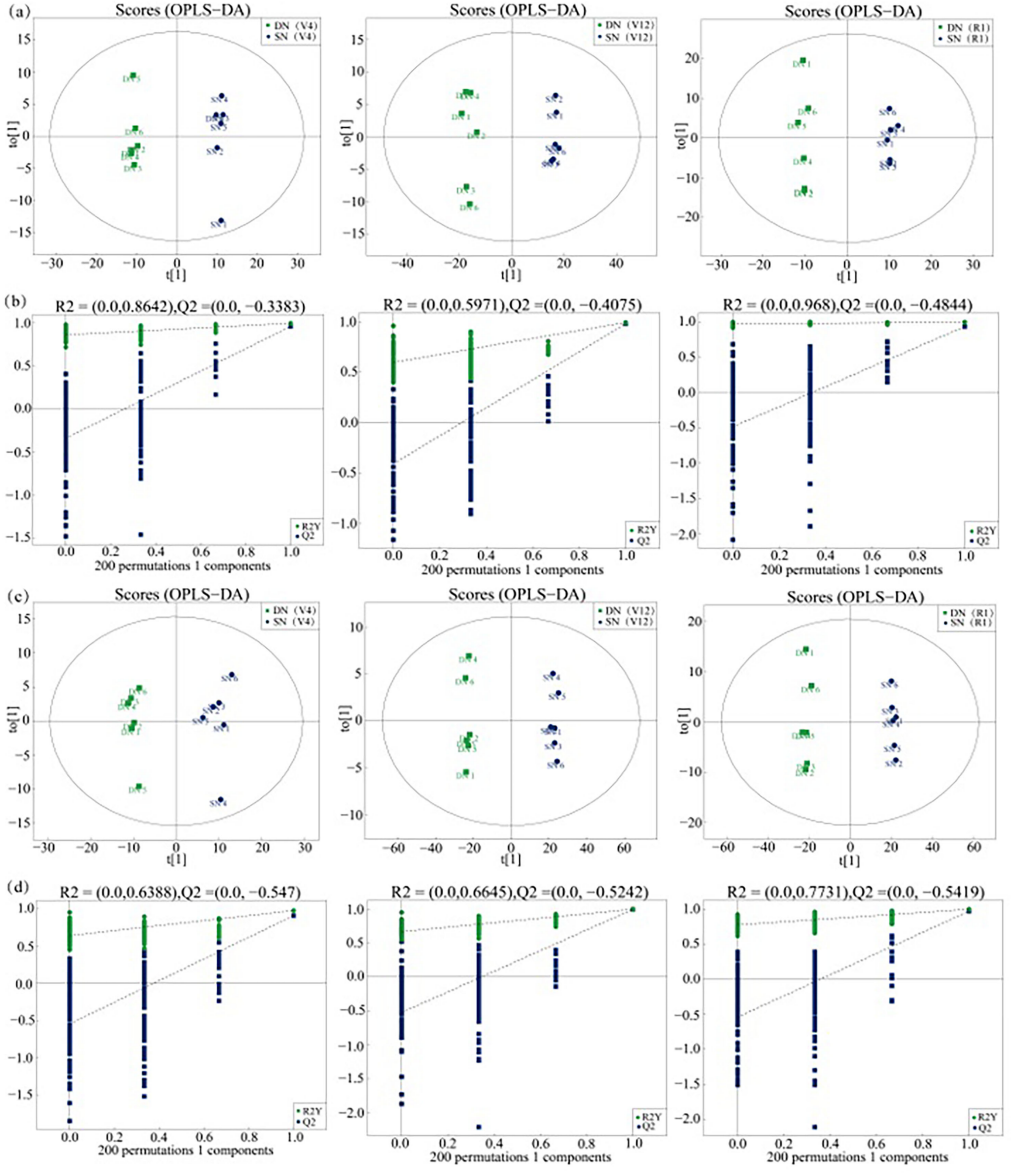
OPLS-DA of metabolites extracted from maize leaves under deficient nitrogen treatment (DN) and sufficient nitrogen treatment (SN). (Note: **(A, B)** show the score plot and the model evaluation plot of DN-SN analysis in ESI^+^ mode, **(C, D)** show the score plot and the model evaluation plot of DN-SN analysis in ESI^-^ mode).

Independent OPLS-DA was performed on the leaf samples of maize plants with DN and SN treatments, as shown in **Figures 3A, C**. The samples of the DN and SN groups were clearly separated along the t[1] axis, achieving suitable classification. In order to avoid overfitting supervised models during the modeling process, a permutation test model was used to ensure the effectiveness of the model. **Figures 3B, D** show the replacement test charts of the OPLS-DA model for these control groups. As the replacement retention gradually decreased, the R^2^ and Q^2^ of the random model also decreased, which indicated that the original model did not have an overfitting phenomenon and the model was robust.

### KEGG pathway enrichment analysis

**Figure 4:**
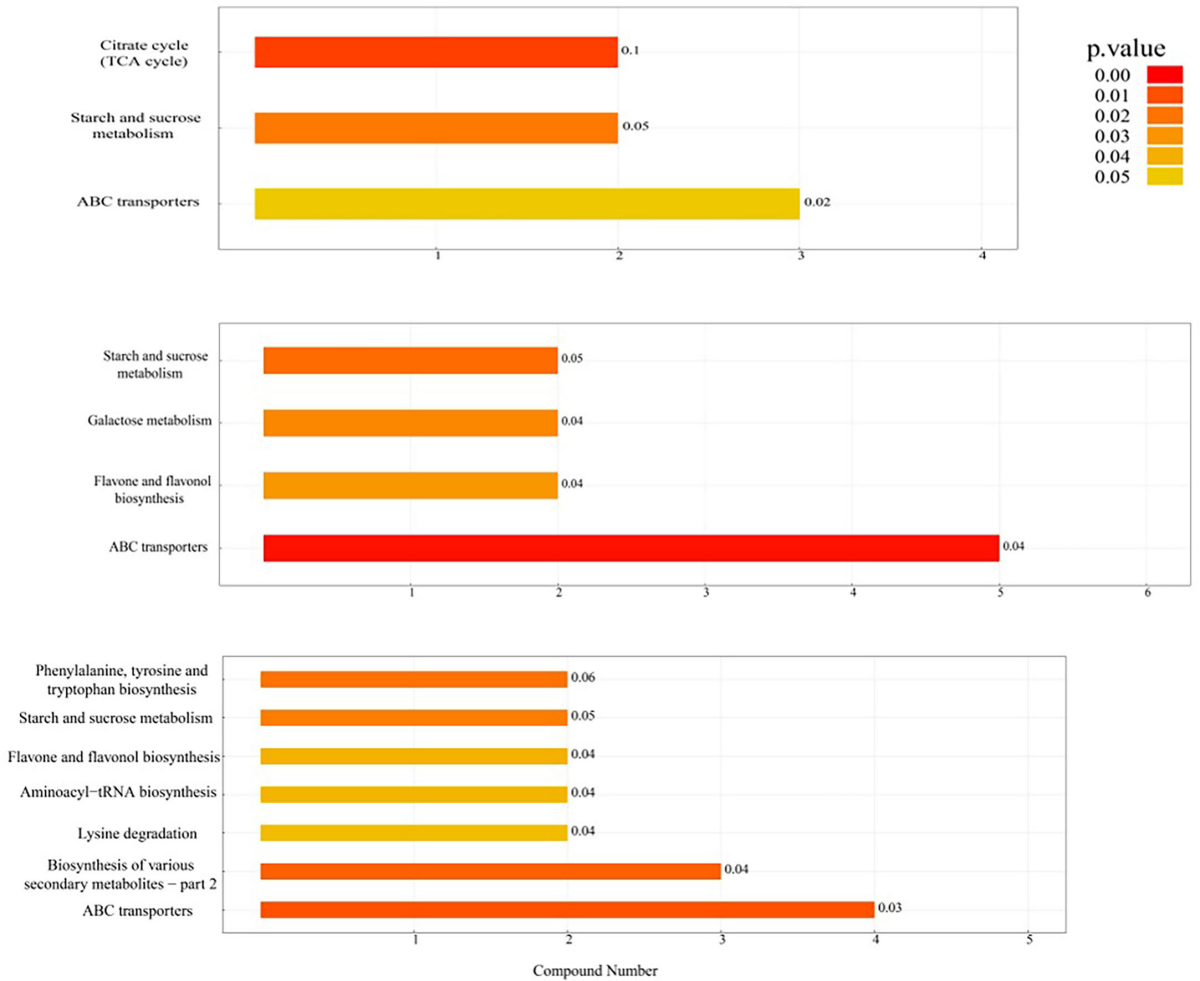
Histogram of KEGG enrichment pathway analysis. The vertical axis in the figure represents each KEGG metabolic pathway, and the horizontal axis represents the number of differentially expressed metabolites contained in each KEGG metabolic pathway. Color represents the average *p* value of enrichment analysis. The darker the color is, the smaller the average *p* value and the more significant the enrichment degree.

As shown in **Figure 4** and **Table 2**, the differential metabolic pathways enriched to the DN/SN comparison group increased as maize grew. Two of these pathways, starch and sucrose metabolism and ABC transporters, changed at different reproductive stages, while the flavone and flavonol biosynthesis pathway changed significantly at V12 and R1 stages. As shown in the figure, under DN treatment, L-malic acid was downregulated and cis-aconitic acid was upregulated in TCA cycle at V4 stage, while sucrose and trehalose were downregulated in starch and sucrose metabolism. This showed that the DN significantly affected sugar metabolism and limited energy storage and utilization. With increasing stress time, at V12 stage, except for sugar metabolism (starch and sucrose metabolism, galactose metabolism), the flavone and flavonol biosynthesis pathway was activated, and secondary metabolites such as flavonoids under DN conditions were significantly upregulated. At R1 stage, DN significantly affected the binding of tryptophan and phenylalanine to tRNA and prevented the formation of the corresponding peptide chains. Additionally, phenylalanine, tyrosine and tryptophan biosynthesis and lysine degradation were affected, mainly affecting tryptophan, phenylalanine, N6, N6, N6-trimethyl-L-lysine and pipecolic acid amino acid synthesis.

**Table 2:** Differential metabolites in KEGG metabolic pathways.

Comparison group	Map Name	Cpd Name	Up/Down
V4 DN/SN	Citrate cycle (TCA cycle)	L-Malic acid/cis-Aconitic acid	down/up
	Starch and sucrose metabolism	Trehalose/Sucrose	down/down
	ABC transporters	Trehalose/Choline/Sucrose	down/down/down
V12 DN/SN	Starch and sucrose metabolism	Trehalose/Sucrose	down/down
	Galactose metabolism	Raffinose/Sucrose	down/down
	Flavone and flavonol biosynthesis	Luteolin/Astragalin	up/up
	ABC transporters	Raffinose/Trehalose/Uridine/Choline/Sucrose	down/down/down/down/down
R1 DN/SN	Phenylalanine, tyrosine and tryptophan biosynthesis	Tryptophan/Phenylalanine	down/down
	Starch and sucrose metabolism	Trehalose/Sucrose	down/down
	Flavone and flavonol biosynthesis	Luteolin/Astragalin	up/up
	Aminoacyl-tRNA biosynthesis	Tryptophan/Phenylalanine	down/down
	Lysine degradation	N6, N6, N6-Trimethyl-L-lysine/Pipecolic acid	down/up
	Biosynthesis of various secondary metabolites - part 2	Tryptophan/Phenylalanine/Podofilox	down/down/down
	ABC transporters	Trehalose/Phenylalanine/Sucrose/2’-Deoxyadenosine	down/down/down/up

Map name indicates the name of metabolic pathway; Cpd Name refers to the name of the metabolite in the KEGG database; up/down indicates the up and down regulation of the metabolite.

### Cluster heatmap analysis of KEGG pathway and identification of differential metabolites

**Figure 5:**
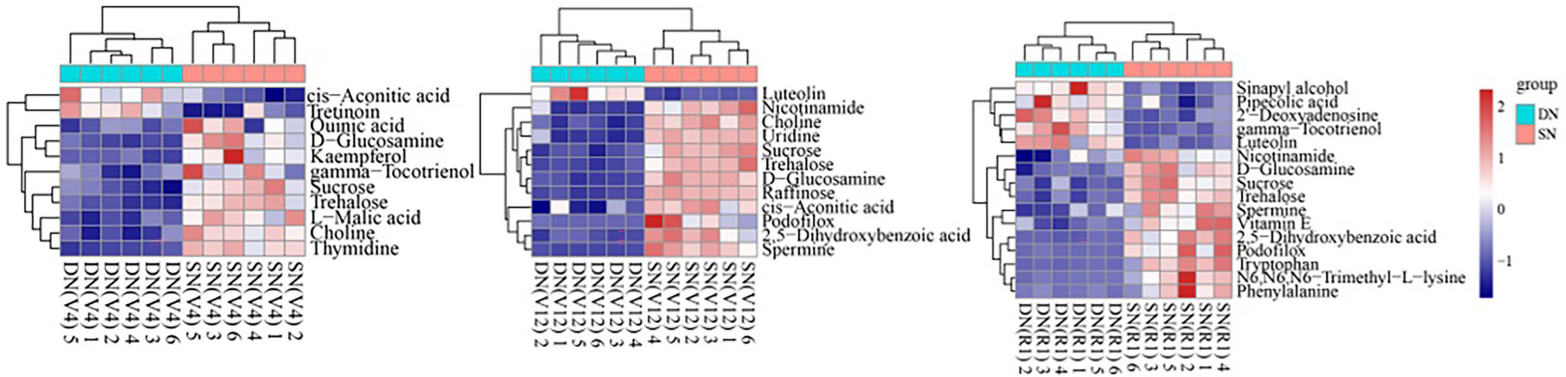
Cluster heatmap of major KEGG pathway metabolites in secondary metabolism. Each row represents a differential metabolite (i.e., metabolites with significant differential expression in the vertical coordinate), and each column represents a set of samples (i.e., sample information in the horizontal coordinate). Red represents significant upregulation, blue represents significant downregulation, and the shade of color indicates the degree of up- or downregulation. Metabolites with similar expression patterns are clustered under the same cluster on the left. The left panel shows the KEGG metabolic pathway involved in the seedling stage comparison group, the middle panel shows the KEGG metabolic pathway involved in the booting stage comparison group, and the right panel shows the KEGG metabolic pathway involved in the anthesis-silking stage comparison group.

In this experiment, the KEGG pathway mapper function was used to label the differential metabolic pathways, and the KEGG metabolic pathways with the number of differential metabolites greater than 5 were selected. The differential metabolites were colored according to the up- and downregulation information, and the differential metabolites in the KEGG metabolic pathway were displayed in the form of a heatmap (**Figure 5**). It was found that the samples treated with two different nitrogen treatments were similar within their respective groups and primarily clustered in the same cluster (**Figure 5**). The metabolites in the same cluster have similar expression patterns and may have similar functions or participate in the same metabolic process or cellular pathway. As shown in **Figure 5**, flavonoids (luteolin and astragalin) showed significant upregulation under DN treatment, and organic oxygen compounds such as sucrose, trehalose and raffinose showed significant upregulation under SN treatment. Specifically, cis-aconitic acid and tretinoin were upregulated under DN treatment at V4 stage, while kaempferol, L-malic acid, sucrose and trehalose were downregulated under DN treatment. At V12 stage, cis-aconitic acid, 2,5-dihydroxybenzoic acid and some organic oxygen compounds were significantly upregulated under SN treatment. In addition to flavonoids accumulated under DN treatment at R1 stage, sinapyl alcohol, gamma-tocotrienol and 2’-deoxyadenosine also showed upregulation under DN treatment, while tryptophan, phenylalanine and N6, N6, N6-trimethyl-L-lysine and other amino acid analogs showed upregulation under SN treatment.

### Response of nitrogen metabolism related pathways to nitrogen levels

**Figure 6:**
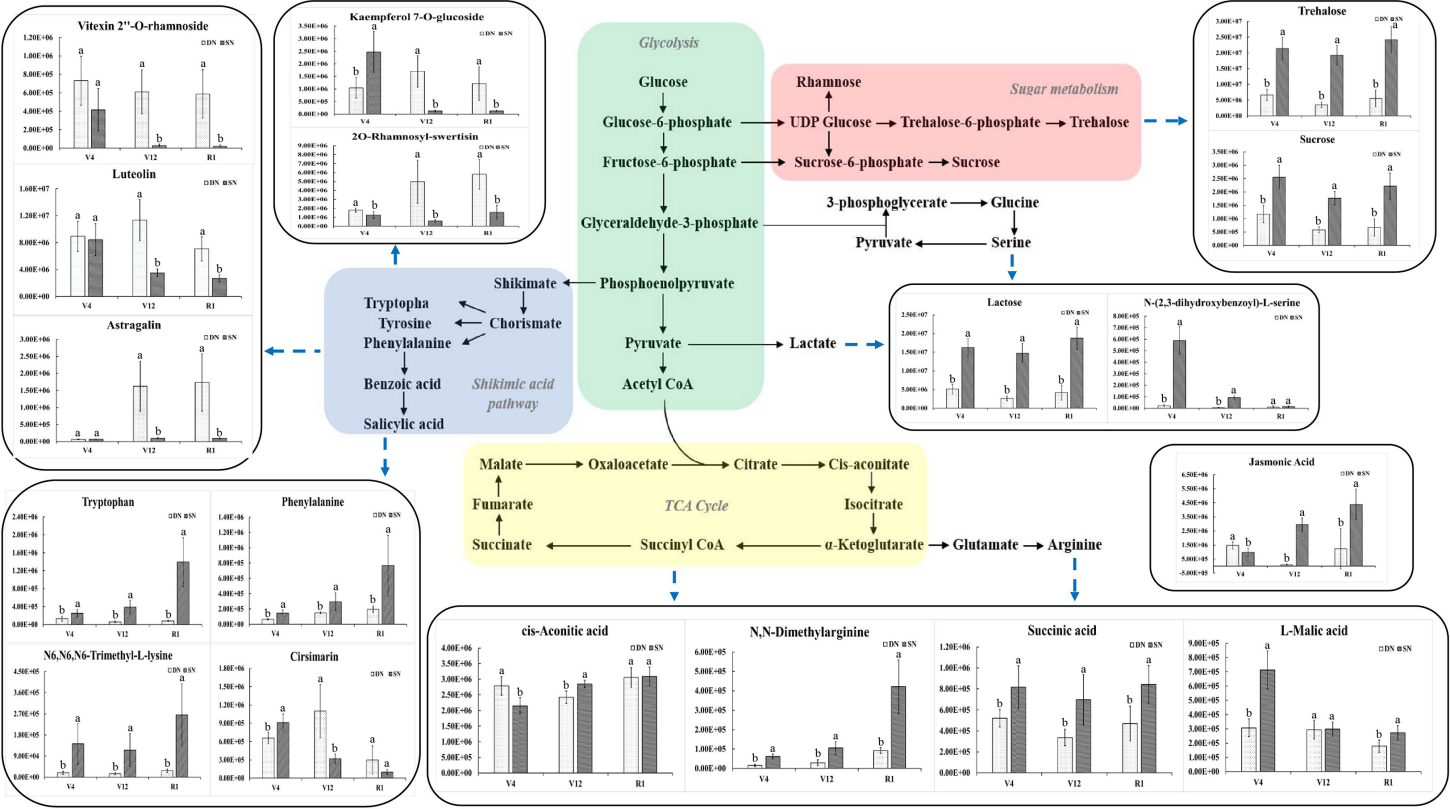
Integration of metabolic pathways and metabolic changes caused by nitrogen stress. The substances marked by blue arrows are the metabolites involved in each metabolic pathway. In the histograms, the x-axis represents different growth stages, and the y-axis represents the average values of relative content. Different letters in the same growth period indicate statistically significant differences at *p* value < 0.05.

**Figure 6** depicts the changes in leaves metabolic pathways and related metabolites induced by nitrogen stress in this study, involving four metabolic pathways, namely, glycolysis, sugar metabolism, the shikimic acid pathway and the TCA cycle. The relative content of trehalose and sucrose produced by the sugar metabolism process was higher under the SN treatment than under the DN treatment and showed a trend of decreasing and then increasing under both treatments as the plants grew and developed, while the pyruvate produced by the glycolytic process produced lactate under the action of lactate dehydrogenase, and the change in its relative content was consistent with the trend of trehalose content.

In this study, four amino acids with significant metabolic differences were identified in maize leaves, namely N, N-dimethylarginine, N6, N6, N6-trimethyl-L-lysine, phenylalanine and tryptophan. The amino acid content was closely related to the amount of nitrogen applied, and the relative content of these four amino acids in maize leaves was relatively high under SN conditions, as shown in **Figure 6**. The relative content of these amino acids increased gradually as plant fertility progressed, and the differences between DN and SN treatments increased (**Figure 6**). Through repeated conditional screening of the differential metabolite data of the comparison groups at three growth stages, some typical flavonoids were found (**Figure 6**). It can be seen from the figure that vitexin 2’’-O-rhamnoside, luteolin, 2O-rhamnosyl-swertisin and other substances under DN treatment have higher relative abundances than SN treatment, which is related to the fact that flavonoids can help improve crop stress resistance. In the DN environment, the relative contents of vitexin 2’’-O-rhamnoside was higher at V4 stage; the relative contents of luteolin and kaempferol 7-O-glucoside were higher at V12 stage than at the other stages; and the relative contents of 2O-rhamnosyl-swertisin and astragalin gradually increased with the growth process (the increase of stress time). The relative contents of vitexin 2’’-O-rhamnoside, luteolin, kaempferol 7-O-glucoside and other substances reached the highest value at V4 stage under SN treatment.

The trends of key intermediates (such as cis-aconitate, succinic acid and malic acid) produced during the TCA cycle were closely related to metabolism. The relative contents of N, N-dimethylarginine, succinic acid and L-malic acid were higher under the SN treatment than under the DN treatment, while the relative content of cis-aconitate was higher under the DN treatment. With the growth process (the increase of stress time), the relative content of N, N-dimethylarginine showed an increasing trend under both conditions, while the relative content of L-malic acid showed a decreasing trend and the relative content of succinic acid showed a decreasing trend followed by an increasing trend. Jasmonic acid is a signaling compound involved in the regulation of plant cell defense and development. It plays an important role in crop growth and development. As seen in **Figure 6**, the relative content of jasmonic acid was higher under the DN treatment at V4 stage, and the relative content of jasmonic acid under the SN treatment conditions was significantly higher at V12 and R1 stages later on with the increase of stress time.

## Discussion

Nitrogen metabolism is essential for plant growth and development (Ashraf et al., 2018). In soils, nitrogen is often a significant factor limiting plant growth, and plants frequently encounter nutrient deficiency in their natural habitats (Krapp et al., 2011; Yang et al., 2017). Based on the field experiment environment, this paper focused on the characteristics and functions related to endogenous nitrogen metabolism in maize leaves at key growth stages. The maize growth response was as expected, nitrogen supply increased plant height, SPAD and leaf dry weight (Sinha et al., 2015; Li et al., 2016; Li et al., 2019). The SPAD values and leaf dry weight under SN treatment were significantly higher than those under DN treatment during the growth process. The enzyme activity of NR and GS under SN treatment was higher than that under DN treatment, which proved that they played important roles in nitrogen assimilation. Studies have shown that appropriate nitrogen fertilizer input can significantly increase the activity of enzymes related to nitrogen metabolism in leaves (Wang et al., 2018), and promote photosynthesis and biosynthesis of organic acids, amino acids, proteins and other nitrogen-containing secondary metabolites in plants (Cao et al., 2020).

At the level of metabolic products, different substances showed different changing trends with the advancement of the growth process. The accumulation of trehalose and sucrose, the glycolytic intermediate lactate and the TCA cycle intermediate succinate in the leaves of maize was limited during the vegetative growth phase (from V4 to V12). In contrast, their relative contents tended to increase during the reproductive growth phase (from V12 to R1). The amino acid pool of plants is characterized by high flexibility and varies between species, cell types, and physiological situations (Millward et al., 2008; Schlüter et al., 2012; Tessari et al., 2016; Kumar et al., 2017). In the environment of nutritional stress, low temperature stress and drought stress, it might stimulate the synthesis of some amino acids (Deng and Lu, 2017; Cao et al., 2020). In this paper, the levels of amino acids such as phenylalanine, tryptophan and N6, N6, N6-trimethyl-L-lysine were significantly increased under SN treatment. This showed that in the field experiment, the total amino acid content of leaves decreased significantly due to nitrogen limitation. The result was consistent with previous research results. Amino acid levels generally decrease under conditions of nitrogen deficiency and conversely increase under conditions of nitrogen saturation (Urbanczyk-Wochniak and Fernie, 2005). Because amino acids were the downstream products of nitrogen metabolism, their abundance increased significantly with the increase in nitrogen levels (Cao et al., 2020).

Plant hormones play an important role in establishing the signaling networks that regulate plant growth and stress-related responses (Ruan et al., 2019). It has been shown that several phytohormones, such as cytokinins, jasmonic acids, and salicylic acids are particularly important during the adaptation to limited nitrogen (Davies and Ribaut, 2017; Mehmood et al., 2021). Jasmonic acid is an endogenous growth regulator present in higher plants and is mainly involved in transmitting external signals to activate the plant’s own defense response system, particularly including the reprogramming of metabolic pathways to initiate and enhance the production of defense compounds against insect herbivores and pathogens (Chen et al., 2019). The mechanisms of action of jasmonic acid are different under different environmental stress factors due to the diversity of plant hormone signals and interactions among different signals (Wang et al., 2020). In this experiment, jasmonic acid, one of the differential metabolites that contributed significantly to the classification of the two nitrogen treatments at V12 and R1 stages. As the duration of nitrogen stress increased, jasmonic acid increased under SN treatment and decreased significantly under DN treatment, indicating that nitrogen stress inhibited its increase, which may also be one of the reasons why nitrogen stress stunted plant growth.

Limited availability of nitrogen fertilizer affects plant growth and metabolism (Schlüter et al., 2012). The different tasks of specialized plant tissues, such as roots, source leaves, young sink leaves, or senescing leaves, would require very different adaptation strategies to low nitrogen (Gifford et al., 2008; Krapp et al., 2011). Flavonoids are polyphenolic secondary metabolites synthesized by plants, and such compounds play a crucial role in protecting plants from oxidative stress and microbial infections (Dias et al., 2021; Caporali et al., 2022). Some studies have pointed out that flavonoids had higher variation in maize than rice, indicating flavonoids are a key constituent of interspecific metabolic divergence. In addition, flavonoids have the ability to withstand heat, frost, and drought and protect plants from UV radiation in sexual reproduction in higher plants, and they are also antipathogenic compounds and key components of interspecific metabolic differences (Deng et al., 2020). Zhang et al. studied the metabolomic changes in wheat under abiotic stress using the UPLC-QTOF technique and found that most of the biomarkers identified in the experiment were flavonoids and their related derivatives, indicating that flavonoid metabolites play an important role in the wheat response to abiotic stress (Zhang et al., 2017). Cao et al. found that the content of some specific secondary metabolites (flavonoids and indole compounds) in the leaves of *Isatis indigotica* seedlings increased under low nitrogen treatment (Cao et al., 2020). The flavonoids identified on summer maize leaves in this experiment, such as kaempferol, luteolin and astragalin, etc., showed significant upregulation under DN treatment and increased with the duration of nitrogen stress, which verified that the plants are stimulated to produce more flavonoids in the natural growth environment due to the severity of nitrogen deficiency.

Metabolites are involved in the metabolic regulation of the entire metabolic network, and a large number of metabolites are involved in primary and secondary metabolic processes (Obata, 2019). Cañas et al. combined metabolomics and enzyme activity analysis to perform correlation and network analyses to determine the regulatory modules involved in the interaction among a large set of leaf physiological characteristics. They proposed that many metabolites and enzyme activities could be used as physiological markers for breeding purposes (Cañas et al., 2017). In this study, some important metabolic pathways in carbon and nitrogen metabolism were significantly affected under DN and SN treatments, and the metabolites involved are indicative in low nitrogen stress. Besides, some secondary metabolites, flavonoids such as kaempferol, Luteolin and astragalin, and hormones such as jasmonic acid, could provide a preliminary basis for the diagnosis of nitrogen stress during the maize reproductive period, and further experiment will focus on targeted validation for the above metabolites and their effects on protein synthesis under nitrogen stress.

## Conclusion

This study showed that the growth of summer maize under nitrogen stress conditions was closely related to metabolic changes. Specifically, (1) nitrogen stress significantly affected carbon metabolism and nitrogen metabolism, the changes of metabolites and their pathways were significantly different between DN and SN treatments, as well as among growth stages. (2) Under nitrogen deficiency, the starch and sucrose metabolism were significantly affected, which happened from V4 stage to R1 stage in maize. (3) With increasing stress time, flavonoid and flavonol biosynthetic stress responses were activated to resist nitrogen deficiency, as evidenced by the significant upregulation of flavonoids such as luteolin and astragalin at V12 and R1 stages under DN treatment. However, jasmonic acid, an endogenous growth regulator, showed significantly higher under SN treatment than under DN treatment. (4) With increasing stress time, the synthesis of tryptophan and phenylalanine and the degradation of lysine were significantly affected. The metabolic synthesis of key amino acids decreased under DN treatment and increased under SN treatment.

## Data availability statement

The raw data supporting the conclusions of this article will be made available by the authors, without undue reservation.

## Author contributions

GS and YL designed the research. GS, YW, CN, MX, LW, and YB participated in data analysis. GS wrote the manuscript. All authors contributed to the article and approved the submitted version.
